# Recurrent selection for wider seedling leaves increases early biomass and leaf area in wheat (*Triticum aestivum* L.)

**DOI:** 10.1093/jxb/eru468

**Published:** 2014-12-11

**Authors:** L. Zhang, R. A. Richards, A. G. Condon, D. C. Liu, G. J. Rebetzke

**Affiliations:** ^1^CSIRO Agriculture Flagship, PO Box 1600, Canberra, ACT 2601,Australia; ^2^Triticeae Research Institute, Sichuan Agricultural University, ChengduChina611130

**Keywords:** Breeding, early vigour, gene action, heritability.

## Abstract

The breeding of wheat with greater early vigour has potential to increase water- and nutrient-use efficiency, as well as to improve weed competitiveness to raise crop yields profitably. Given that wheat is inherently conservative in its early growth, a sustained breeding effort was initiated to increase genetically seedling leaf area in developing novel high vigour germplasm. A recurrent selection programme was initiated by intercrossing a genetically diverse set of 28 vigorous wheat lines identified globally. These were intercrossed at random and S_1:2_ progeny with the largest leaf 1 and 2 widths were intermated to develop new populations for assessment of early growth. This procedure was repeated for up to 60 segregating families per cycle across six cycles over 15 years. Thirty random S_1:2_ progeny were retained from each cycle and seed-increased together to produce seed for early vigour assessment in multiple sowings. The most vigorous wheat seedlings were identified in later cycles, with some lines producing more than double the leaf area and biomass of elite commercial wheat varieties. Phenotypic selection for greater leaf width was associated with a realized significant (*P*<0.01) linear increase per seedling of 0.41mm per cycle (+7.1%) for mean leaf width, and correlated linear increases in total leaf area and biomass of 4.48cm^2^ per cycle (+10.3%) and 10.8mg per cycle (+5.3%), respectively. Genetic gains in widths of leaves 2 (+8.4%) and 3 (+11.5%) were significantly (*P*<0.01) greater than for leaf 1 (+5.3%). Selection for greater leaf width was associated with linear increases in coleoptile tiller leaf area, small curvilinear increases in leaf 1 length, and reductions in numbers of leaves and mainstem tillers. Genetic variances were large and heritabilities high for leaf width and total leaf area in each cycle, but reduced linearly in size with selection across cycles. Coupling diverse germplasm with a simple, inexpensive, and repeatable selection process has confirmed the value of recurrent selection in developing uniquely vigorous wheat germplasm for use as parents in commercial breeding.

## Highlight

Long-term, S2 recurrent selection for seedling leaf width has produced vigorous wheat germplasm with potential for genetic improvement of water-use efficiency, weed competitiveness, and nutrient-use efficiency.

## Introduction

A large portion of the world’s rainfed wheat (*Triticum aestivum* L.) is grown in Mediterranean-type climates characterized by cool, wet winters and hot, dry summers (Ward *et al.*, 2008). Reliable rainfall for cropping in these environments occurs during the cool, winter months, with rainfall rapidly declining together with the onset of increasing vapour pressure deficit (VPD) during spring and into summer. In turn, crops generally have adequate soil moisture during the vegetative period and must make efficient use of this soil water particularly when water is plentiful early in the season (Siddique *et al.*, 1990). Wheat breeders have been successful in maintaining genetic gain near 0.5% per year through yield-based selection and particularly by fine-tuning genotypic adaptation through changes in phenology and plant height to increase harvest index (Perry and d’Antuono, 1989). These simple genetic changes have contributed to genetic gain in water-limited yield potential (Siddique *et al.*, 1990), highlighting the opportunity and capacity to identify and exploit new additional genetic diversity to improve gain in water-use efficiency (WUE; Richards *et al.*, 2002).

Development of a biological framework has potential to identify limitations to crop performance for a target environment or environment type. Physiological constraints to growth and yield can be addressed genetically to enrich gene pools for traits where favourable alleles may be at a low frequency (Araus *et al.*, 2002; Richards *et al.*, 2002). Greater early vigour contributing to more rapid early leaf area development is a trait of particular relevance to Mediterranean environments (López-Castañeda and Richards, 1994). Genotypes producing greater early vigour grow more rapidly after germination to increase leaf area and biomass up to anthesis. A more rapid leaf area development shades the soil surface to reduce soil evaporation and increase crop WUE significantly (López-Castañeda and Richards, 1994). Greater growth during the cooler winter months when VPDs are low also increases transpiration efficiency (Fischer, 1979). Ward *et al.* (2008) increased soil nitrogen to increase early growth and thereby slow soil evaporation to increase WUE in low-to-intermediate in-season rainfall environments of Western Australia. Increasing early growth has potential for increasing uptake of soil nitrogen (Pang *et al.*, 2014) and phosphorus (Ryan *et al.*, 2014) to improve crop nutrient-use efficiencies and provide better competition with weeds (Coleman *et al.*, 2001). More vigorous crops intercept more light to maximize crop growth rates and biomass particularly with late sowing or for environments where crop duration is shorter (Takahashi and Gotoh, 1996; Regan *et al.*, 1997).

Barley generally performs well in Mediterranean and other dryland environments where its more rapid leaf area and biomass development contributes to its greater WUE when compared with wheat (López-Castañeda and Richards, 1994). Wheat is inherently conservative in its early growth (López-Castañeda *et al.*, 1996), though crop growth modelling has previously indicated that greater early vigour has the potential to increase average grain yields by 8–10% in Mediterranean environments typical of parts of the Australian wheat belt (Condon *et al.*, 2004). Earlier studies assessing small genotypic increases in early vigour have demonstrated the potential to increase biomass and grain yield with selection for vigour in Mediterranean environments (e.g. Whan *et al.*, 1991; Botwright *et al.*, 2002). Yet while there is compelling evidence for the benefits of early vigour in winter cereals, there is little evidence of targeted breeding for this trait in crop improvement programmes.

There is potential to increase early vigour through selection for large grain size, although this does rely on harvesting from environments characterized by favourable conditions through grain filling. In contrast, breeding and selection for greater early vigour has a number of benefits in development of wheat germplasm and new varieties with intrinsically more rapid leaf area development. To understand the underlying drivers of differences in vigour among temperate cereals, López-Castañeda *et al*. (1995, 1996) examined factors associated with the 2-fold greater leaf area of barley compared with wheat despite an identical seed size. They found that specific leaf area (SLA) and embryo size accounted for most of the variation in vigour among temperate cereals as there were no differences in relative growth rates or net assimilation rates (López-Castañeda *et al.*, 1995, 1996). They proposed that selection for the width of the first few seedling leaves should integrate embryo size and SLA, and this would be a simple way to screen and select for high early vigour. Rebetzke and Richards (1999) later established that seedling leaf width was highly heritable and had a high genetic correlation with total leaf area in wheat during the vegetative stage. Richards and Lukacs (2002) also established the importance of both embryo size and SLA in determining vigour among wheat lines.

Genotypic variation for early vigour in wheat is under control of many genes of largely additive genetic effect (Rebetzke *et al*., 2004 2008*a*, 2014). Genetic diversity for greater vigour has been identified across a broad range of germplasm sources, and, where pedigree information is available, this diversity and the alleles contributing to it should be unrelated (Rebetzke and Richards, 1999; Richards and Lukacs, 2002). The potential exists to accumulate favourable alleles from diverse sources through intra- and interpopulation development breeding strategies. One such strategy, recurrent selection, is based on repeated selection of superior individuals followed by their intermating, thereby allowing for efficient recombining of desirable alleles from diverse gene pools to maximize genetic variance and subsequent genetic gain (Hallauer and Darrah, 1985). Where heritabilities are high, phenotypic recurrent selection has proven to be a rapid and efficient means for accumulating favourable alleles from a diverse range of genetic sources (e.g. Kenworthy and Brim, 1979; Delzer *et al.*, 1995). Attempts to increase early vigour through either improved establishment or development of larger seedlings have been restricted to allogamous species where controlled intermating and seed production is simpler. Here genetic gains for vigour of up to 10% per cycle have been demonstrated across a range of species including pasture legumes (Klos and Brummer, 2000; DeHaan *et al.*, 2001) and various pasture grasses (e.g. Faulkner *et al.*, 1982; Jefferson *et al.*, 2001). Recurrent selection from pools representing diverse genetic sources has also been demonstrated for improved establishment in wheat (Gul and Allan, 1976) and pearl millet (Lynch, 1994), and for increased seedling growth of maize (Mock and Bakri, 1976) and sorghum (Bacon *et al.*, 1986) in cool soils.

The early vigour phenotype and morphological factors contributing to early leaf area development are clearly defined in wheat (Rebetzke and Richards, 1999). The opportunity exists to identify and pyramid favourable alleles from unrelated wheat germplasm in the development of elite parental lines with greater early vigour for use in commercial breeding. The base population for a phenotypic recurrent selection mating scheme was generated from intermating unrelated, vigorous wheats and full-sib families from a high vigour biparental population. Elite families for greater leaf width were identified and intermated over six cycles and progeny retained for assessment. This paper reports on the development and implementation of an S_1:2_ phenotypic recurrent selection programme for genetic improvement of early vigour in wheat, and the assessment of genetic gain for leaf width and correlated response for early leaf area and biomass across multiple environments.

## Materials and methods

### Population development

**Fig. 1.**
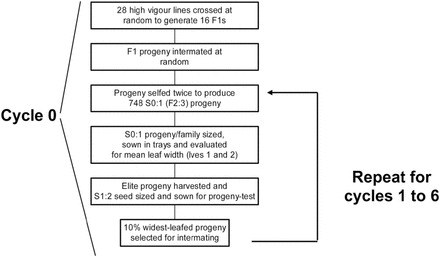
Procedure used in generating the base and subsequent cycles in the generation of the recurrent selection population.

A large and globally representative set of >2000 wheat genotypes were assembled and screened under controlled environment conditions for leaf 1 and 2 width, and early leaf area (see Rebetzke and Richards, 1999; Richards and Lukacs, 2002). The most vigorous of these genotypes were selected for intermating in the development of a recurrent selection population and included: (i) eight vigorous lines derived from a genetically diverse 10-parent, composite cross (Quail *et al.*, 1989); (ii) 13 F_4_-derived, full sibs from a cross of the two vigorous donors, Jing Hong and Kharchia (Richards and Lukacs 2002); and (iii) seven genetically unrelated varieties and breeding lines. Collectively, these parental lines were assembled to develop the base population (Fig. 1). Briefly, the JingHong/Kharchia full sibs (ii) were crossed and then mated at random among themselves to produce 22 F_1_ progeny. Other sources of vigour represented by (i) and (iii) lines were intercrossed at random to develop another 16 F_1_ progeny. The total set of 38 F_1_s were self-pollinated to produce S_0_ progeny which underwent a further round of self-pollination without selection to produce S_0:1_ progeny.

Between 40 and 50 S_0:1_ seed free of any visible damage were sampled from each of the 38 populations and sized to a restricted range of 40–45mg. These seed were sown following Rebetzke *et al.* (2004) into paper-lined, wooden seedling trays (600×300×120mm) containing a fertile, soil-based potting mix, and then grown outdoors during the winter of 1996 in Canberra Australia in an unreplicated experimental design augmented with multiple replicates of two control entries—a commercial parent ‘Janz’ and high vigour germplasm ‘Vigour18’. Non-destructive measurements were made of the breadth of leaves 1 and 2 (i.e. mean leaf width) after the majority of plants reached ~3.4 leaves and all second leaves had fully expanded. Approximately half the population producing the largest mean leaf width were retained and grown under favourable conditions to maturity, whereupon seed was harvested (Fig. 1). Two S_1:2_ seed were sized to a range of 40–45mg for each line and sown and assessed the following winter in a replicated row–column design using the method described above. The two largest mean leaf width S_1:2_ lines from each population were transplanted into pots and grown to anthesis for subsequent crossing and generation of a new cycle. Pedigrees were maintained throughout and crossing was restricted to minimize mating of closely related progeny. Altogether, ~80 crosses were made to generate 80 new cycle 1 populations. This process was repeated six times over the next 12 years toward the development and generation of cycle 6 progeny (Fig. 1). Selection intensity was high, varying across cycles from 1.9% to 2.3%. Long-term average mean temperature for the coldest winter months when phenotyping is 5.7 °C.

### Seed production

A random subset of S_1:2_ progeny from each cycle (selected and unselected) were stored together in a –18 °C freezer. In 2012, seed of 30 lines were sampled at random from each cycle and sown into a large (25cm) pot containing a fertile potting mix. All cycles were recovered except cycle 2 which was lost with long-term storage. In addition to the recurrent selection-derived lines, a sample of the original parents, eight modern and representative spring and winter wheat varieties, and the barley (*Hordeum vulgare* L.) variety Franklin and triticale (×*Triticosecale* Wittmack) variety Currency were sown into large pots. The lines were randomized and replicate pots provided for the original parents, commercial spring wheats, and barley. All pots were placed into a cool glasshouse and grown under favourable conditions to produce good quality seed. Time to flowering and plant height were measured on each line. Heads were harvested at maturity and then carefully hand-threshed before weighing and storing in a –18 °C freezer until used for experimentation.

### Cycle assessment

Three S_1:2_ seed were sized to a range of 45–50mg for each of 30 random lines in each cycle and commercial check varieties. In three sowings (sowing dates of 13 May, 19 July, and 5 October 2013) seed were sown to a depth of 20mm into wooden seedling trays (600×300×120mm) containing a fertile, compost-based potting mix. In a separate sowing (sowing date of 12 August), seed were sown at 20mm depth into 50cm deep, 10cm diameter PVC tubes containing a 75% compost, 25% sand blend. Each sowing contained three replicates in an AR(1)×AR(1) row–column spatial design developed using DiGGer software (Coombes, 2009). Here lines from the same cycle were constrained from appearing multiple times in the same row or column of each replicate (William Bovill, personal communication). Seed germinated and seedlings subsequently were grown under well-watered conditions in trays and deep tubes placed outdoors in an open, sunlit area, and the experiment was repeated across four winter and spring sowing dates (13 May, 19 July, 12 August, and 5 October) in 2013. Average temperatures for May, June, July, August, September, and October in 2013 were 9.4, 7.8, 7.6, 8.6, 12.0, and 12.9°C, respectively. Seedlings were grown outdoors and supplied with adequate nutrition and irrigation up to when ~80% of plants reached the 4.2-leaf stage when all plants were sampled. Measurements were made on all plants of the numbers of leaves on the mainstem, length, and breadth of leaves 1, 2, and 3, and, where fully expanded, leaf 4. Mainstem and coleoptile tillers were also counted. Plants were cut at soil level and then placed into a fan-forced dehydrator heated to 70 °C for drying and then weighing. Leaf area was calculated from the products of length and breadth of all leaves, correcting for leaf shape with a factor of 0.8 (Rebetzke and Richards, 1999). Leaf area ratio was then calculated for the mainstem as total leaf area÷(total stem+leaf biomass). Coleoptile and mainstem tiller leaf area were calculated from the product of dry weight and leaf area ratio.

### Statistical analysis

Data were analysed statistically after first checking for normality and error variance heterogeneity across sowings. Residual plots revealed a non-random distribution for mainstem and coleoptile tiller characters (frequency, biomass, and leaf area) requiring arcsine data transformation. Combined analyses of variance and covariance over sowing dates were then performed for all traits using the SAS mixed linear models procedure MIXED (Littell *et al.*, 1996). Models included fitting of row and column within tray covariance factors, and a single factor for seed weight. Broad-sense heritabilities (H) were calculated on an entry-mean and single-plant basis after Holland *et al.* (2003), and genetic correlations were estimated after Holland (2006). Cycle 0 to 6 means based on all lines in each cycle were used in estimating the slope and subsequent genetic progress across cycles for all traits. Genetic progress over the entire recurrent selection programme was calculated relative to trait mean performance in the parental population. Unless otherwise indicated, statistical significance is given for an alpha of 0.05.

## Results

### Environments

Environmental variation for measured seedling characteristics is presented in Table 1. Total leaf area and biomass were smallest for the early May 13 sowing due to the earlier harvest for this sowing (~375 °Cd). In turn, leaf 4 was not fully elongated and few if any seedlings produced a fifth leaf compared with all other sowings (data not shown). The earlier harvest also resulted in a significantly reduced mainstem tiller frequency and size (Table 1). The remaining sowings were all harvested at an approximately similar growth stage (~450 °Cd). Total leaf area was similar for all three later sowings, but mainstem leaf area (excluding tillers) was smallest for the August 12 sowing.

**Table 1.** Environment means for early vigour characteristics measured on 271 lines representing multiple cycles from a recurrent selection population and commercial controls

Environment	Mean leaf width^*a*^ (mm)	Leaf 1 width (mm)	Leaf 1 length (mm)	No. of mainstem leaves	Mainstem tiller number	Mainstem tiller leaf area (cm^2^)	Coleoptile tiller frequency (%)	Coleoptile tiller leaf area (cm^2^)	Total plant and mainstem leaf areas (cm^2^)^*b*^	Total plant biomass (mg)
Sowing 1 (13 May 2013)^*c*^	7.72	7.14	99	3.78	0.13	1.25	63	3.47	51.2 (46.4)	177
Sowing 2 (19 July 2013)^c^	7.50	7.00	94	4.41	0.56	7.34	78	6.57	64.2 (50.4)	275
Sowing 3 (12 August 2013)^*d*^	6.78	6.42	79	4.12	1.85	16.38	87	6.08	65.5 (40.3)	248
Sowing 4 (5 October 2013)^*c*^	7.18	6.43	107	4.40	0.23	3.23	53	4.00	64.4 (57.2)	278
Average LSD^*e*^	0.20	0.21	4	0.10	0.04	0.91	6	0.87	3.2 (2.2)	23

^*a*^ Mean leaf widths of leaves 1 and 2.

^*b*^ Total plant and mainstem only (in parentheses) leaf areas.

^*c*^ Tray studies.

^*d*^ Tube studies.

^*e*^ Average LSD for comparisons across sowings.

Sowings producing greater mean biomass and leaf area reflected production of longer and wider leaves, and increased numbers of mainstem leaves at harvest. Comparing across sowing dates, leaf 1 width and mean leaf width was greatest for the two earlier sowings (May and July) and especially for the earliest mid-May sowing. Leaf 1 length was also significantly longer for the earliest sowing, contributing to a significantly greater leaf 1 area (data not shown). The longest leaves were produced in the later sowing 4, contributing to this sowing’s greater total leaf area despite the narrower leaf widths.

### Genotypic variation

Seed germination and early growth were considered excellent for control entries and progeny from all cycles (data not shown). A full statistical analysis was subsequently undertaken for all genotypes across sowings. Individual seed weight was fitted as a linear factor and for most traits was significantly different from zero (Table 2). Resulting slopes were commonly positive (data not shown). This suggests that despite efforts to restrict the range in seed size, small increases in seed weight were associated with a general increase in the size of individual traits (e.g. leaf width and coleoptile tiller leaf area). However, while significant, their general effect was small, with a 10mg increase in seed weight associated with an average mean leaf 1 width and length increase of 0.23mm and 3.1mm, respectively (Table 2). Increasing seed weight was associated with significant reductions in numbers of mainstem leaves but not mainstem tiller frequency or leaf area. On the other hand, increases in seed weight contributed to increased frequency and size of coleoptile tillers (Table 2).

**Table 2.** Variance components (±SEs), heritabilities, and genetic correlations with total leaf area and biomass for early vigour characteristics measured on recurrent selection-derived and control cereal genotypes assessed across four sowing dates

Entry	Mean leaf width (mm)	Leaf 1 width (mm)	Leaf 1 length (mm)	No. of mainstem leaves	Mainstem tiller frequency (*n*)	Mainstem tiller leaf area (cm^2^)	Coleoptile tiller frequency (*n*)	Coleoptile tiller leaf area (cm^2^)	Total plant leaf area (cm^2^)	Total plant biomass (mg)
Entry	1.09±0.09**	0.69±0.06**	148±14**	0.047±0.005**	1.55±0.50**	40±11**	3.7±0.5**	2.38±0.55**	95±11**	1042±147**
Entry×sowing	0.03±0.01*	0.02±0.01ns	37±16*	0.008±0.002**	3.23±0.86**	62±18**	2.1±0.6**	3.72±0.81**	21±5**	333±128**
Residual	0.22±0.01**	0.23±0.01**	75±4**	0.052±0.003**	17.9±0.94**	368±19**	12.6±0.7**	15.9±0.85**	92±5**	2670±145**
Seed weight	0.023±0.007**	0.031±0.006**	0.31±0.10**	–0.011±0.002*	–0.009±0.031 NS	–0.17±0.14 NS	1.1±0.3**	0.09±0.03**	0.711±0.110**	3.706±0.479**
Heritability^*a*^	98 (81)	97 (73)	91 (57)	88 (43)	60 (13*)	46 (9^†^)	73 (22)	51 (11)	88 (45)	77 (26)
Total leaf area (*r*_g_)	0.90**	0.88**	0.86**	–0.68**	–0.82**	–0.21*	0.34**	0.44**	–	0.97**
Total biomass (*r*_g_)	0.88**	0.85**	0.73**	–0.44**	–0.81**	–0.23*	0.43**	0.38**	0.97**	–

^*a*^ Entry-mean heritability (single-plant heritability in parentheses).

*,**denotes that the parameter is statistically different from zero at *P*=0.05 and 0.01, respectively.

^†^ *P*=0.10.

The genotypic variances were large and statistically significant for plant leaf area and component traits including mean leaf width and length, mainstem and coleoptile tiller frequency, and leaf area (Table 2). The interaction of entry with sowing date was commonly small to intermediate in size, except for mainstem and coleoptile tiller frequency and biomass, and to a lesser extent total plant biomass. The interaction variance was particularly small for leaf 1 width and mean leaf width, being approximately 1/30th the size of their respective genotypic variances. In turn, entry-mean heritabilities were largest for leaf widths and lengths, numbers of leaves, and total plant leaf area, and smallest for mainstem and coleoptile tiller leaf area (Table 2). Single-plant heritabilities across traits were consistent in ranking with entry-mean heritabilities and were particularly high for leaf 1 and mean leaf widths. Across all entries and sowings, genotypic increases in leaf area and biomass were strongly correlated with increases in leaf width and to a lesser extent leaf length (Table 2). Total leaf area was correlated with total biomass, while increases in numbers of mainstem leaves and both mainstem tiller frequency and size were associated with reductions in total leaf area and biomass.

**Fig. 2.**
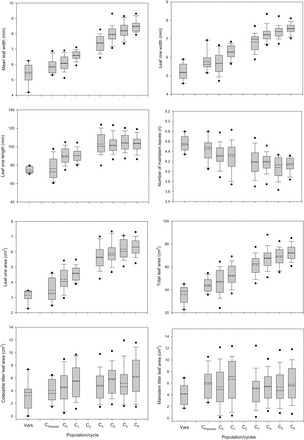
Box-plots summarizing mean leaf width and other measured characteristics averaged across all sowings for wheat varieties (‘Vars’) and lines in each cycle (parental population to cycle 6) of the leaf width recurrent selection programme. The dots represent extreme line values, the whiskers the 5th and 95th percentiles, the boxes the 25th and 75th percentiles, and the solid and dashed lines the median and mean, respectively, for each character. Cycle 2 lines were accidently discarded during long-term seed storage.

The control entries varied significantly for all traits, with triticale and barley varieties producing significantly larger biomass than tested wheat varieties (Table 3). This size difference largely reflected the wider leaves, particularly of the barley. Spring wheat varieties were more vigorous than winter varieties, again reflecting their wider leaves. This was particularly true of the spring wheat varieties Axe and Westonia which were among the most vigorous of commercial wheats tested (data not shown). That aside, individual lines were identified in cycles 4, 5, and 6 with more than double the leaf area and biomass of the most vigorous commercial wheat (Fig. 2).

**Table 3.** Cycle and entry means for early vigour characteristics measured on recurrent selection-derived and control cereal genotypes assessed across four sowing dates

Entry	Mean leaf width (mm)	Leaf 1 width (mm)	Leaf 2 width (mm)	Leaf 3 width (mm)	Leaf 1 length (mm)	No. of mainstem leaves	Mainstem tiller frequency (*n*)	Mainstem tiller leaf area (cm^2^)	Coleoptile tiller frequency (*n*)	Coleoptile tiller leaf area (cm^2^)	Total plant (mainstem) leaf area (cm^2^)	Total plant biomass (mg)
Cycle means												
Parental generation	5.89	5.61	6.14	7.58	77	4.44	0.47	5.70	0.57	3.99	53.9 (44.2)	213
Cycle 0	6.09	5.76	6.42	8.02	90	4.32	0.50	5.36	0.68	4.29	56.3 (46.6)	210
Cycle 1	6.60	6.30	6.90	8.89	91	4.31	0.54	6.66	0.71	5.21	64.5 (52.6)	221
Cycle 3	7.39	6.86	7.92	10.76	102	4.17	0.33	5.07	0.66	4.87	71.5 (61.6)	267
Cycle 4	7.98	7.25	8.70	11.99	102	4.17	0.34	5.07	0.65	5.79	78.6 (67.7)	282
Cycle 5	8.24	7.40	9.08	12.62	104	4.08	0.33	5.39	0.65	5.14	79.1 (68.6)	280
Cycle 6	8.40	7.60	9.39	12.95	103	4.13	0.32	5.35	0.68	6.04	83.8 (72.4)	297
Average LSD^*a*^	0.10	0.10	0.10	0.16	3	0.11	0.07	1.01	0.07	0.79	2.9 (2.1)	13
Cycle linear	0.41**	0.30**	0.52**	0.87**	3.21*	–0.05**	–0.03*	–0.12	–0.01 NS	0.21**	4.48**	10.8**
(*r* ^2^)	(0.97)	(0.96)	(0.98)	(0.97)	(0.78)	(0.87)	(0.73)	(0.19)	(0.11)	(0.59)	(0.96)	(0.92)
Control entries												
Spring wheat varieties	5.37	5.23	5.36	6.65	71	4.54	0.67	6.91	0.67	4.19	47.1 (36.0)	167
Winter wheat varieties	5.00	4.94	5.02	6.55	65	4.36	0.53	4.45	0.36	1.55	32.9 (26.9)	156
Barley	6.76	6.92	7.05	7.99	51	5.01	0.68	5.56	0.21	0.78	41.5 (35.2)	204
Triticale	6.22	5.69	6.94	8.55	86	4.56	0.75	8.01	0.19	1.88	53.5 (43.6)	213
Average LSA (with cycles)^*b*^	0.31	0.26	0.39	0.47	8	0.22	0.20	2.6	0.31	1.98	5.2 (4.4)	42
Average LSD (vars)^*c*^	0.78	0.65	0.69	0.79	13	0.41	0.39	4.5	0.57	4.25	10.1 (9.1)	72

^*a*^ Average LSD for comparing among cycle means.

^*b*^ Average LSD for comparing between control and cycle means.

^*c*^ Average LSD for comparing between control means.

### Direct selection for mean leaf width

Genotypic variance was large and significant (*P*<0.01) for all early vigour characteristics measured in all cycles (Table 3; Fig. 2). When compared with the commercial wheats, mean performance of all cycles was significantly greater for leaf 1, 2, and 3, and mean leaf widths, total plant leaf area, and biomass (Table 3). The mean leaf widths of Australian spring and winter varieties were on average significantly smaller than that of lines ZL49B, ZL59B, Jing Hong, Kharchia, and V743 used as parents in generating the cycle 0 population (Table 3; Fig. 2). Commercial wheats tended to produce more mainstem leaves and tillers, although tiller leaf area was not statistically different (Table 3).

**Fig. 3.**
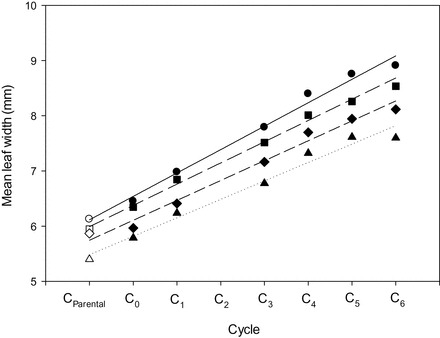
Relationship between cycle number and mean leaf width measured in four environments: sowing 1 (circles; *y*= –6.53+0.44 *x*, *r* ^2^=0.97, *P*<0.01); sowing 2 (squares; *y*=6.41+0.39 *x*, *r* ^2^=0.98, *P*<0.01); sowing 3 (diamonds; *y*=5.87+0.34 *x*, *r* ^2^=0.97, *P*<0.01); and sowing 4 (triangles; *y*=6.02+0.38 *x*, *r* ^2^=0.95, *P*<0.01). Note that cycle 2 lines were accidently discarded during long-term seed storage.

Recurrent selection for mean leaf width was successful in producing linear increases in mean leaf width across six cycles of S_1:2_ selection (Table 3). The coefficient of determination was high (*r* ^2^=0.97), indicating a strongly linear increase in mean leaf width despite some evidence of a curvilinear reduction (*P*>0.05) from cycles 3 to 6 (Figs 2, 3). Examination of mean leaf width for individual sowing dates confirmed linear increases in three of the four sowings, with evidence of a significant reduction in cycle 6 mean leaf width for sowing 4 (Fig. 3). Yet despite the small change in later cycles, slopes remained fairly consistent, ranging from 0.34 to 0.44mm per cycle (Fig. 3). Averaged across all sowing dates, the estimated slope of 0.41mm per cycle (7.1%) for mean leaf width (Table 3) indicated an average leaf width increase of 2.46mm, or 42%, across all cycles of selection. This compares with slopes of 0.31 (+5.3%) and 0.52 (+8.4%) mm per cycle for leaves 1 and 2, respectively. The greatest increase in leaf width was associated with leaf 3, which increased 0.87mm (+11.5%) per cycle (Table 3). A number of cycle 6 lines produced leaf 4 widths in excess of 30mm which compared favourably with the narrow leaf 4 widths of the commercial varieties (average of 13.8mm) and high vigour parents (average of 16.9mm) used in generating the recurrent selection programme. The previously most vigorous line identified in the early vigour breeding programme, Vigour 18, produced among the widest leaf widths of all lines in cycle 0 (6.49mm). However, by cycle 3, all recurrent selection derivatives were as vigorous as Vigour 18 (Fig. 2).

Genotypic distributions for mean leaf width were continuous in each cycle, consistent with polygenic control for this trait (Fig. 2). For example, a number of cycle 0 lines were sampled producing leaf 1 and mean leaf widths smaller than that of the smaller parental lines. A large range in mean leaf width was observed among lines, but selection into later cycles was associated with reduced genetic variance, as indicated by reductions in both heritability and genotypic coefficients of variation (Table 4). Reductions were also observed for leaf 1 width, but it appears that reduction in genetic variance may be greater for this leaf than for leaves 2 and 3 (data not shown).

**Table 4.** Range in genotype means, narrow-sense heritability, and genetic coefficients of variation for early vigour characteristics measured in each of three cycles of recurrent selection for greater mean leaf widthGenotypic correlations with plant leaf area and biomass are indicated for recurrent selection lines.

Cycle	Parameter	Trait										
		Mean leaf width (mm)	Leaf 1 width (mm)	Leaf 1 length (mm)	No. of mainstem leaves	Mainstem tiller leaf area (cm^2^)	Coleoptile tiller leaf area (cm^2^)	Total plant leaf area (cm^2^)	Total plant biomass (mg)	100-grain weight (g)	Plant height (cm)	Days to anthesis
Cycle 0	Range	5.09–7.11**	3.88–6.69**	75–105**	3.7–4.8**	0.10–12.2*	0.3–9.23*	22.7–64.7**	105– 316**	1.91–5.89**	55–110**	56–74**
	h^2^	0.88	0.88	0.82	0.62	0.26	0.57	0.83	0.39	0.92	0.87	0.89
	GCV	7.9	8.5	7.7	4.5	9.0	39	15.1	9.1	23.2	10.1	8.3
Cycle 3	Range	5.92–8.79**	5.73–7.93**	79–128**	3.5–4.7**	0–13.5*	0.4–12.4*	43.7–80.0**	128–351*	2.30–5.97**	58–99**	55–74**
	h^2^	0.78	0.65	0.79	0.65	0.16	0.36	0.68	0.42	0.86	0.84	0.92
	GCV	6.9	5.1	8.6	4.9	4.5	29	10.3	9.6	19.7	7.8	8.7
Cycle 6	Range	7.91–9.44**	6.95–8.31**	85–121*	3.8–4.3*	0.3–11.9*	0.7–11.7*	57.9–87.3*	219–382*	2.64–5.72**	56–103**	55–72**
	h^2^	0.48	0.31	0.58	0.39	0.11	0.60	0.19	0.15	0.83	0.85	0.90
	GCV	3.8	2.5	6.3	1.8	2.3	45	3.4	2.5	20.4	9.2	8.1
*r*_g_ total leaf area		0.93**	0.89**	0.85**	–0.73**	–0.34**	0.31**	–	0.91**	0.15*	0.13*	0.06 NS
*r*_g_ total biomass		0.76**	0.78**	0.73**	–0.54**	–0.30**	0.40**	0.91**	–	0.11 NS	0.08 NS	0.03 NS

*,**indicates that correlations with leaf area and biomass are significantly different from zero at *P*=0.05 and 0.01, respectively.

NS indicates that correlations with leaf area and biomass are not significantly different from zero at *P*=0.05.

### Correlated effects with selection

**Fig. 4.**
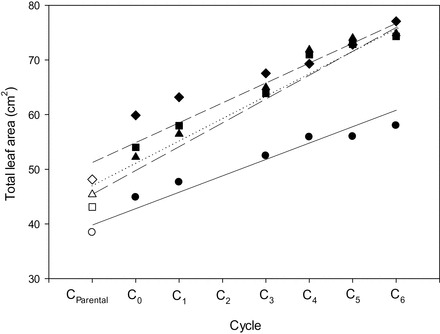
Relationship between cycle number and mean total leaf area measured in four environments: sowing 1 (circles; *y*=37.7+3.19 *x*, *r* ^2^=0.93, *P*<0.01); sowing 2 (squares; *y*=41.8+5.16 *x*, *r* ^2^=0.94, *P*<0.01); sowing 3 (diamonds; *y*=48.5+4.24 *x*, *r* ^2^=0.93, *P*<0.01); and sowing 4 (triangles; *y*=41.8+5.26 *x*, *r* ^2^=0.95, *P*<0.01). Note that cycle 2 lines were accidently discarded during long-term seed storage.

Selection for increases in mean leaf width was associated with significant changes across a range of traits (Table 3; Fig. 2). Both leaf 1 and total leaf areas increased linearly, with evidence for a continued increase in later cycles (Fig. 2). The increase in total leaf area was consistent across cycles in all four sowing dates, although change in leaf area was greatest for sowing 2 and 4 where leaf number was greatest (Fig. 4). Leaf 4 area was greatest in these sowings, contributing to the increased total leaf areas and particularly for later cycles of selection (data not shown). Leaf 1 length increased curvilinearly in early cycles but then plateaued, with little or no change in length for cycles 4–6. Numbers of mainstem leaves decreased with selection for greater leaf width. The change was small (0.05 leaves or 1.1% per cycle) but was strongly linear (*r* ^2^=0.87) (Table 3). Consistent with a reduction in leaf number and increased leaf length was the linear reduction in the frequency of mainstem tillers. Again the change was small but significant, with reductions of 6.4% per cycle. Mainstem tiller size as leaf area was unchanged with selection (Table 3; Fig. 2). However, coleoptile tiller frequency and leaf area increased significantly with selection for mean leaf width (Table 3).

Genotypic variation for recurrent selection lines commonly covaried with changes in total leaf area and biomass consistent with the size and nature of relationships reported in Table 2 for all genotypes. Selection for wider leaves was associated with a reduction in the genetic variance for leaf 1 length and numbers of leaves, whereas mainstem and coleoptile tiller leaf area were unchanged (Table 4). Assessment of agronomic traits indicated that selection for wider leaves was associated with small average increases in seed size (*r*_g_=0.15, *P*<0.05), whereas plant height and time to flowering were unchanged (Table 4). In turn, there was a small reduction in the genetic variance for seed size, but variation in plant height and time to flowering was similar across all cycles.

Among traits, changes in mainstem and coleoptile tiller frequency were genotypically uncorrelated [*r*_g_=0.04; non-significant (NS)], whereas coleoptile tiller and mainstem tiller leaf areas were moderately negatively correlated (*r*_g_= –0.42, *P*<0.01). Genotypic variation in mainstem and coleoptile tiller frequency and size was not associated with differences in leaf 1 length (*r*_g_= –0.07 to 0.11; NS), but numbers of mainstem leaves and leaf 1 length were strongly, negatively correlated (*r*_g_= –0.66, *P*<0.01). Mainstem tiller number and numbers of mainstem leaves were themselves positively correlated (*r*_g_=0.28, *P*<0.01).

## Discussion

Recurrent selection is a population improvement method widely used in selection of allogamous species such as maize. The benefits where the target phenotype is heritable and inexpensive to measure are many and include (i) rapid cycling and subsequent accumulation of favourable alleles from many parents into a single superior genotype; (ii) the capacity for increased recombination and the breaking of repulsion-phase linkage blocks; and (iii) the opportunity to select simultaneously for negatively correlated traits (Bailey and Comstock, 1976; Hallauer and Darrah, 1985). However, the need for emasculation and hand pollination, and production of few seed restricts the potential for use of recurrent selection in autogamous species such as wheat. Nonetheless, where used in soybean (e.g. grain protein; Brim and Burton, 1979) and wheat (e.g. kernel size; Busch and Kofoid, 1982), phenotypic recurrent selection has been effective in integrating alleles from many potential sources in long-term genetic improvement of complex traits. The success of these examples reflected a reliable (heritable) and simple to measure phenotype. Similarly, a diverse range of seemingly genetically unrelated sources for early vigour were identified from a large global wheat survey (Rebetzke and Richards, 1999; Richards and Lukacs, 2002). Together with knowledge that early vigour is under complex genetic control with alleles distributed throughout the genome (Rebetzke *et al*., 2008*a*, 2014), and given known pedigree information that parental sources were unrelated by descent, a recurrent selection programme was commenced aimed at accumulating alleles for early vigour. Underpinning this effort was previous knowledge that the mean width of leaves 1 and 2 was under strong additive genetic control and had high narrow-sense heritability, and that the additive genetic correlations for mean leaf width and total leaf area were high (Rebetzke and Richards, 1999). Taken together, it was expected that longer term genetic gain for early vigour could be achieved via indirect selection for mean leaf width in spite of the higher selection intensities used.

Large effective population sizes were utilized and sib×sib matings were minimized to ensure both short- and longer term genetic gain despite the higher selection intensities used throughout. Genetic gain for mean leaf width was strongly linear, although there was some suggestion of reduced gain in cycle 6 particularly in a late spring (5 October) sowing (Fig. 3). The maintenance of genetic gain over a sustained period has contributed to the development of wheat lines producing more than double the seedling leaf area and biomass of the most vigorous commercial wheats. The use of S_1:2_ selection coupled with a pedigree-based crossing scheme allowed for the combining and selecting of genes for greater early vigour from genetically diverse sources, thereby contributing to a sustained linear genetic gain. Assuming no epistasis, the genetic variance among S_1:2_ families includes 1σ^2^_A_+0.125σ^2^_D_+1.250D_1_+0.187D_2_ where σ^2^_A_ and σ^2^_D_ are the additive and dominance genetic variances, respectively, D_1_ is the covariance between additive and homozygous dominance effects, and D_2_ is the variance among homozygous dominance effects (Cockerham, 1983). Thus there is opportunity to exploit additive effects in selection among non-inbred families, and, if heritability is high, opportunity for genetic gain through selection within S_1_ families. Genetically, S_1:2_ selection partitions more of the total additive genetic variance between families (and thereby reduces variance within families) to increase confidence in selecting for additive genetic effects that are transmitted from parent to progeny (Burton and Carver, 1993). The estimated gains for mean leaf width of 7% per cycle were high, but were comparable with estimates of genetic gain for other highly heritable traits reported in the literature (e.g. kernel size in wheat; Busch and Kofoid, 1982). Selection for mean leaf width was closely associated with linear increases in widths of leaves 1, 2, and 3, with genetic gain being greater for leaf 2 (8.4% per cycle) than leaf 1 (5.3% per cycle), and even greater for leaf 3 (11.5% per cycle) which was not under selection. Indeed, response in leaf 3 width was 2-fold greater than for leaf 1. Much of the tissue for leaf 3 is not present at germination (López-Castañeda *et al.*, 1996) which infers that potential growth of leaf 3 is contingent on the growth and assimilates produced by earlier leaves.

Selection for greater mean leaf width was associated with significantly greater seedling leaf area and biomass. As for mean leaf width, genetic progress for these two traits was strongly linear across cycles. The genetic correlations for leaf width and both total seedling leaf area and biomass were strong and, together with the high heritability for mean leaf width, contributed to a high correlated genetic gain for these genetically complex traits. Similar correlated genetic response for leaf width and both total leaf area and biomass was reported previously for wheat (Rebetzke and Richards, 1999). No selection was made for greater leaf length. However, there was evidence for a small increase in leaf 1 length from the base population (cycle 0) to cycle 1, whereas changes in leaf 1 length in later stages of selection were not significant. In turn, leaf length was not an important contributor to changes in total leaf area in later stages of selection despite the modest genetic correlation for the two traits.

Numbers of leaves and tillers (both mainstem and coleoptile) can contribute to genetic changes in early leaf area development in wheat. However, it was reported previously that genotypic increases in both numbers of leaves and mainstem tillers were associated with reductions in early vigour (Rebetzke and Richards, 1999). Indeed, selection for wider leaves and greater total seedling leaf area was associated with a linear reduction in both numbers of leaves and mainstem tillers across cycles. Similarly, production of longer leaves across genotypes was closely related to the production of fewer leaves and to a lesser extent fewer tillers. Recurrent selection for seedling tiller number in other species has confirmed reductions in seedling biomass with divergent selection into high tillering families (e.g. Altai wildrye; Jefferson *et al.*, 2001). Despite the reduction in numbers of mainstem tillers, there was not a corresponding reduction in tiller leaf area (or biomass; data not shown), reflecting the observation that the fewer tillers initiated in earlier cycles were small and contributed little to early leaf area development. Mainstem tillers are borne later and may therefore contribute little to early ground cover (Rebetzke and Richards, 1999). However, they are important in final spike number (Mitchell *et al.*, 2013). Coleoptile tiller frequency remained largely unchanged with selection for mean leaf width. However, coleoptile tiller leaf area increased, contributing to increases in total leaf area. Coleoptile tillers emerge well in advance of the first mainstem tiller and so can contribute significantly to early leaf area development (e.g. Liang and Richards, 1995; Rebetzke and Richards, 1999; Richards and Lukacs, 2002). Strong additive genetic control suggests the potential for progress in selecting for more frequent and larger coleoptile tillers (Rebetzke *et al.*, 2008*a*). However, moderate sized negative genetic correlations for coleoptile and mainstem tiller leaf areas suggest that efforts to increase simultaneously both toward greater leaf area and final spike number may be challenging.

Given the contribution of leaf length to phyllochron and tiller number (Kirby *et al.*, 1985) in a highly synchronous species such as wheat, emphasis should be given to controlling leaf length when selecting for greater early vigour. Indeed, the recurrent selection study has confirmed the need for a focused contribution of greater mean leaf width over leaf length in selecting for greater early vigour owing to its (i) higher single plant heritability and (ii) strong genetic correlation with early leaf area and biomass in unreplicated, early generation assessment. Further, selection for leaf width permits the maintenance of increased numbers of leaves and tillers, both of which are reduced in selection for increased leaf length.

The high genetic gain estimates of 7.1% per cycle for mean leaf width were consistent with previous reports for establishment or seedling dry weight in other crops. For example, Twamley (1974) reported a 10–15% increase in seedling dry weight of birdsfoot trefoil (*Lotus corniculatus* L.) following three cycles of phenotypic recurrent selection. However, here and elsewhere (e.g. Dehaan *et al.*, 2001), increased seed weight was a major contributor to improved early growth, and so many studies conclude that selection for seed size should be a breeding objective for greater early vigour. Indeed, establishment and early vigour are greater in larger seeded wheats (e.g. Lafond and Baker, 1986; Aparicio *et al.*, 2002; Richards and Lukacs, 2002; Royo *et al.*, 2006). However, large maternal covariances can reduce the potential for increased grain size (e.g. Thiede, 1998), particularly in environments where grain number is high (and grain size smaller) or where carbohydrate production is impaired to reduce final grain size (e.g. with disease, high temperature, or terminal droughts; Rebetzke *et al.*, 2008*b*). For the study reported herein, growing parents under the same common environmental conditions, and then standardizing to a common grain size, the aim was too reduce the potential for maternal factors in confounding selection for alleles conditioning greater early vigour. While seed size is commonly linked to greater vigour, recurrent selection for improved establishment and early biomass in reed canarygrass (*Phalaris arundinacea* L.) was associated with a small reduction in seed weight (Casler and Undersander, 2006).

Despite the potential benefits for greater early vigour in cereals, there has been little evidence of targeted breeding for this trait in cereal improvement programmes. In part, this may reflect the lower heritabilities and reduced selection response for seedling leaf area and biomass (Rebetzke and Richards, 1999). It may also reflect the confounding influence of grain size and other environmentally induced maternal effects on genetic differences in early seedling development (Richards and Lukacs, 2002). Another possibility is that the vigour source used in earlier studies was not especially vigorous and that trait introgression via backcrossing was not successful in recovery of all vigour-based alleles. The recurrent selection programme has demonstrated the capacity for germplasm development and particularly parent-building in accumulating many alleles for use in breeding. The leaf width phenotype is reliable and repeatable, with little evidence for genotype×environment interaction to slow genetic progress. There was little change in plant height or time to flowering with selection, indicating that it is possible to recover combinations of reduced plant height (Botwright *et al.*, 2005) and phenologies appropriate to a target environment.

In this breeding context it is important to consider the possible advantages and disadvantages of enhanced vigour in yield improvement programmes and also circumstances where enhanced vigour may be neutral for yield. Greater seedling vigour is likely to contribute most to yield when the potential leaf area index of a crop is restrained by factors such as: growing season rainfall (particularly in Mediterranean environments), season duration, conservation farming practices, weediness, poor nutrition, and salinity. In these environments, lines with a faster early leaf area development should result in a greater biomass and yield than lines with low vigour. In these less favourable environments there may also be the potential for crop management practices to enhance leaf area and biomass. For example, additional nitrogen and/or increased sowing rate can also increase crop growth and may be used as a surrogate for genetic increases in vigour. However, these involve additional costs to the farmer. A genetic solution to enhance vigour is preferred as this does not involve any additional costs to the farmer. There are some possible disadvantages of greater early vigour. One is the greater transpiration and hence crop water use associated with increased leaf area when the top soil is dry, as this may deplete soil water and lead to lower yields. However, in environments where the topsoil is typically moist prior to canopy closure, this early depletion of soil water is unlikely to occur and greater vigour typically results in higher yields (e.g. Ward *et al.*, 2008). An important factor contributing to greater early vigour is a high SLA (López-Castañeda *et al.*, 1995; Richards and Lukacs, 2002). SLA is inversely related to net assimilation rate in temperate cereals probably because of the reduced investment in photosynthetic machinery per unit leaf area (Richards, 2000). This should not be a problem before canopy closure because of the increased leaf area achieved by a high SLA, but it may be a hindrance after canopy closure. However, this may be small as SLA declines as anthesis approaches (Rawson *et al.*, 1987). Another possible disadvantage of enhanced seedling vigour may occur if the wide early leaves responsible for the vigour are maintained in all leaves up to flowering, as this may negate the achievement of a desirable canopy architecture. This also seems unlikely as there appears to be little relationship between the dimensions of the first formed leaves and the last leaves just prior to flowering in temperate cereals (Richards and Rebetzke, personal observations). Environments where greater seedling vigour is likely to be neutral to yield is in crops with either a longer duration or crops grown under favourable conditions and which can achieve a high leaf area index.

In conclusion, the value of phenotypic S_1:2_ recurrent selection for greater leaf width in development of more vigorous wheat germplasm has been demonstrated. Elite vigorous germplasm are now being used in population development of wheats with improved WUE and weed competitiveness.
